# Isoliquiritigenin Nanoemulsion Preparation by Combined Sonication and Phase-Inversion Composition Method: In Vitro Anticancer Activities

**DOI:** 10.3390/bioengineering9080382

**Published:** 2022-08-10

**Authors:** Jianman Wang, Hongjin Chen, Tao Guo, Ping Yue, Tianbao Qian, Xiangyu Zeng, Yali Luo, Jiangmin Li, Lijing Teng, Qingyu Liu, Liang Hong, Zijiang Yu, Zuquan Hu

**Affiliations:** 1 Key Laboratory of Infectious Immune and Antibody Engineering of Guizhou Province, Engineering Research Center of Cellular Immunotherapy of Guizhou Province, School of Basic Medical Sciences/School of Biology and Engineering, Guizhou Medical University, Guiyang 550025, China; 2 Department of Cardiovascular Surgery, Affiliated Hospital of Guizhou Medical University, Guiyang 550009, China; 3 Guizhou Institute of Precision Medicine, Affiliated Hospital of Guizhou Medical University, Guiyang 550009, China; 4 Translational Medicine Research Center, Guizhou Medical University, Guiyang 550025, China; 5 Immune Cells and Antibody Engineering Research Center of Guizhou Province, Key Laboratory of Biology and Medical Engineering, Guizhou Medical University, Guiyang 550025, China

**Keywords:** isoliquiritigenin, nanoemulsion, sonication, phase-inversion composition (PIC)

## Abstract

Isoliquiritigenin (ILQ) has a number of biological activities such as antitumor and anti-inflammatory effects. However, biomedical applications of ILQ are impeded by its poor aqueous solubility. Therefore, in this research, we prepared a novel ILQ-loaded nanoemulsion, i.e., ILQ-NE, which consisted of Labrafil^®^ M 1944 CS (oil), Cremophor^®^ EL (surfactant), ILQ, and phosphate-buffered saline, by employing a combined sonication (high-energy) and phase-inversion composition (low-energy) method (denoted as the SPIC method). The ILQ-NE increased the ILQ solubility ~1000 times more than its intrinsic solubility. It contained spherical droplets with a mean diameter of 44.10 ± 0.28 nm and a narrow size distribution. The ILQ loading capacity was 4%. The droplet size of ILQ-NE remained unchanged during storage at 4 °C for 56 days. Nanoemulsion encapsulation effectively prevented ILQ from degradation under ultraviolet light irradiation, and enhanced the ILQ in vitro release rate. In addition, ILQ-NE showed higher cellular uptake and superior cytotoxicity to 4T1 cancer cells compared with free ILQ formulations. In conclusion, ILQ-NE may facilitate the biomedical application of ILQ, and the SPIC method presents an attractive avenue for bridging the merits and eliminating the shortcomings of traditional high-energy methods and low-energy methods.

## 1. Introduction

**Figure 1:**
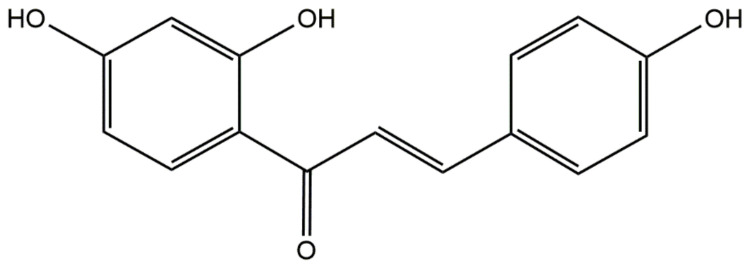
The structure of isoliquiritigenin.

Isoliquiritigenin (ILQ, Figure 1) is a chalcone-type flavonoid compound that can be extracted from the roots of *Glycyrrhiza uralensis*. It possesses multiple biological activities, including antimicrobial, antitumor, and anti-inflammatory effects [1,2,3]. For instance, ILQ can suppress *Staphylococcus xylosus* growth by inhibiting imidazole glycerol phosphate dehydratase (IGPD), which can be utilized as a target protein to treat infections caused by *S. xylosus* [4]. In addition, ILQ can suppress breast cancer via affecting NF-κB signaling and the PI3K–Akt pathway, inhibiting p38 expression and downregulating arachidonic acid-metabolizing enzymes [1]. ILQ can inhibit gastric cancer stemness, modulate the tumor microenvironment, and suppress tumor growth through downregulating glucose-regulated protein 78 [2]. ILQ is easily soluble in some organic solvents and alkaline aqueous solutions, but it is difficult to dissolve in water. In addition, due to its poor solubility and quick elimination, ILQ has poor absorption in vivo and low bioavailability, which hinders its further in vivo application. To increase the solubility of ILQ, various delivery systems, including self-micro-emulsifying drug delivery system (SMEDDS) [3], nanosuspensions [5], polymeric micelles [6], D-α-tocopheryl polyethylene glycol 1000 succinate (TPGS)-modified proliposomes [7], nanostructured lipid carriers [8], lipid-polymer hybrid nanoparticles [9], and mesoporous silica nanoparticles [10], have recently been developed. For example, the orally bioavailable ILQ SMEDDS was developed, which significantly improved the solubility, stability, permeability, and other properties of ILQ [3]. The solubility of ILQ after encapsulation into polymeric micelles was 232 times higher than its intrinsic solubility [6]. The solubility and bioavailability of ILQ were also significantly improved by the TPGS-modified proliposomes [7]. However, the application of these delivery systems has been hindered by some obstacles, including poor storage stability, low chemical stability, inadequate bioactivity, and complex preparation processes.

Among these delivery systems, nanoemulsions have attracted great attention owing to merits such as their small droplet size, high stability, and ease of processing. Nanoemulsions (oil-in-water (O/W) type) are kinetically stable dispersions that typically consist of oil, a surfactant (and possibly a cosurfactant), and water, with droplet sizes ranging from 10 to 500 nm [11]. Nanoemulsions can be made using either high-energy or low-energy technologies in the past. To make microscopic droplets of nanoemulsions, high-energy methods use mechanical disruptive forces generated by specialized mechanical devices such as sonicators, microfluidizers, and high-pressure valve homogenizers [12]. The advantage of high-energy methods in preparing nanoemulsions include suitability for producing nanoemulsions using a wide variety of surfactants and oils, the capability of generating small droplets using a small amount of surfactant [13,14]. However, high-energy methods also have shortcomings such as high cost due to expensive equipment, and the possible destruction of bioactive compounds that are sensitive to temperature elevation, such as ILQ [15]. The use of low-energy technologies is another conventional method of creating nanoemulsions. They rely on surfactant–oil–water combinations that spontaneously form droplets under the appropriate environmental conditions [15]. Examples of low-energy processes include the phase-inversion composition (PIC), phase-inversion temperature (PIT), and spontaneous emulsification [16]. Low-energy techniques have a number of benefits, including low costs and minimal damage to bioactive chemicals [15,17]. Nevertheless, the types of surfactants and oils that can form nanoemulsions through low-energy methods are limited, and a large amount of surfactant is needed for low-energy methods to create small droplets, leading to safety concerns [18,19]. The combination of high-energy methods and low-energy methods might bridge the merits and eliminate the shortcomings of the two methods. Recently, approaches combining high-energy methods and low-energy methods for producing nanoemulsions have been reported [20,21]. However, systemic investigations on novel effective preparation methods that combine the advantages of high-energy methods and merits of low-energy methods, i.e., methods that generate tiny droplets with low surfactant concentration and prevent degradation of active components like ILQ, are still rare.

Therefore, in this research, we explored the possibility of producing ILQ nanoemulsions using an approach that combines sonication (high-energy method) and PIC (low-energy method), denoted as the SPIC method, to improve the aqueous solubility, chemical stability, in vitro release, and cancer cell-killing ability of ILQ. Phosphate-buffered saline (PBS, pH 7.4) was used as an aqueous phase to improve the solubility of ILQ in an aqueous physiological environment. Compared with other high-energy methods, such as high-pressure homogenizers and microfluidizers, sonication provides advantages such as low maintenance costs owing to easy machine cleaning and operation [22]. Amongst various low-energy methods, the PIC method provides the prominent advantage of convenient operation since it is performed at room temperature. Therefore, the SPIC method that combines sonication and PIC, offers an effective and facile approach to produce nanoemulsions. We optimized the preparation process of ILQ nanoemulsions via investigating the effects of the stirring speed, bath sonication water height, bath sonication time, and ILQ concentration on the droplet size and storage stability, and obtained the optimized ILQ nanoemulsion, abbreviated as ILQ-NE. Afterwards, we evaluated the visual appearance, droplet size distribution, morphology, ultraviolet (UV)–visible light absorption spectrum, and fluorescence emission spectrum. Subsequently, we investigated the physical stability, chemical stability, in vitro release profile, cytotoxicity to cancer cells, and cellular uptake of ILQ-NE. Producing small droplets without ILQ destruction, the SPIC method presents an intriguing avenue for improving the solubility of bioactive compounds that are easily damaged.

## 2. Materials and Methods

### 2.1. Materials

Isoliquiritigen (purity ≥ 98.0%) was purchased from Aladdin Biochemical Technology Co., LTD (Shanghai, China). Labrafil^®^ M 1944 CS (Oleoyl macrogol-6 glycerides) was purchased from Gattefosse (Saint Periest Cedex, France). Cremophor^®^ EL (PEG-35 castor oil, CAS number 61791-12-6) was obtained from BASF (Ludwigshafen, Germany). Dimethyl sulfoxide (DMSO), Tween 80, RPMI 1640 medium and foetal bovine serum (FBS) were purchased from Chaoyuan Zhicheng Biotechnologies Co., LTD (Guiyang, China). Dialysis membranes (molecular weight cut-off 3500 D) were purchased from Aoyi Biotechnology Co., LTD (Guiyang, China). KH_2_PO_4_ and NaCl were purchased from Fengchuan Chemical Reagent Technology Co., LTD (Tianjin, China). KCl was obtained from Comere Chemical Reagents Co., LTD (Tianjin, China). Na_2_HPO_4_·12H_2_O was purchased from Zhiyuan Chemical Reagent Co., LTD (Tianjin, China). Deionized water was produced by ELGA VEOLIA (Veolia Water Solutions & Technologies, Shanghai, China).

### 2.2. Solubility Studies

The solubility of ILQ in various excipients, including oils and surfactants, was measured using UV-visible light spectrophotometry. Excess ILQ (50 mg) was added to 2 mL of each excipient. Then, the mixture was stirred on a multipoint magnetic stirrer (CJB-S-10D, Yuming Instrument equipment Co., LTD, Shanghai, China) at room temperature for 24 h (750 r min^−1^). After that, the mixtures were centrifuged at 8000 r min^−1^ for 30 min to remove the precipitate. The supernatants were diluted with acetonitrile. Finally, the absorbance at 372 nm (i.e., absorption peak value of ILQ) was recorded using a UV-visible spectrophotometer (A360, Aoyi Instruments Co., LTD, Shanghai, China). The solubility was calculated after establishment of a standard curve for the relationship between absorbance at 372 nm and ILQ concentration.

### 2.3. Optimization of Nanoemulsion Preparation Process

The SPIC method was used to prepare nanoemulsions. Unless otherwise stated, the nanoemulsion was produced as follows. First, a vial containing the organic phase consisting of oil (Labrafil^®^ M 1944 CS, 0.18 g), a surfactant (Cremophor^®^ EL, 0.12 g), and ILQ (24 mg) was partially immersed in the water and sonicated for 20 min (750 r min^−1^) using an ultrasonic cleaning machine (SB-4200D, Xinzhi Biotechnology Co., LTD, Ningbo, China). The length of the ultrasonic cleaning machine was 32.5 cm, and the width was 26.5 cm. Afterwards, the vial was put on a multipoint magnetic stirrer (CJB-S-10D, Yuming Instrument equipment Co., LTD, Shanghai, China) and stirred for 20 min (750 r min^−1^). Then, the aqueous phase (2.7 mL PBS, pH 7.4) was added into the organic phase dropwise with a 1 mL injector while it was under constant stirring (750 r min^−1^) at a rate of 1 drop per second. After the addition of the aqueous phase, stirring was continued for 30 min. The preparation process was carried out at room temperature. In some experiments, specific parameters were varied to investigate their effects on the diameter of the droplet prepared by the SPIC method.

#### 2.3.1. Stirring Speed

In order to explore the influence of different stirring speeds on droplet size, the stirring speed was set at 500, 750, 1000, or 1250 r min^−1^ at room temperature.

#### 2.3.2. Bath Sonication Water Height

The influence of bath sonication water height on droplet size was investigated by sonicating the organic phase (ILQ, Labrafil^®^ M 1944 CS and Cremophor^®^ EL) at different heights (12, 13, 14, 15, or 16 cm) in an ultrasonic cleaning machine with a length of 32.5 cm and a width of 26.5 cm for 20 min before the titration of the aqueous phase.

#### 2.3.3. Bath Sonication Time

The influence of the bath sonication time on droplet size was investigated by setting different ultrasonic times (0, 10, 20, or 30 min).

#### 2.3.4. ILQ Concentration

The effect of the ILQ concentration on the droplet size of ILQ-NE was investigated by changing the amount (0, 12, 24, or 36 mg) of ILQ added to a 3 mL system, obtaining ILQ concentrations of 0, 4, 8, and 12 mg mL^−1^.

### 2.4. The Optimized ILQ Nanoemulsion (ILQ-NE) Preparation

First, an organic phase containing oil (Labrafil^®^ M 1944 CS, 0.18 g), a surfactant (Cremophor^®^ EL, 0.12 g), and ILQ (12 mg) was sonicated with an ultrasonic cleaning machine (SB-4200D, Xinzhi Biotechnology Co., LTD, Ningbo, China) for 20 min (750 r min^−1^). Then, the organic phase was stirred for 20 min (750 r min^−1^). Finally, the aqueous phase (2.7 mL PBS, pH 7.4) was added into the organic phase dropwise using a 1 mL injector under constant stirring (750 r min^−1^) at a rate of 1 drop per second. After the drops were added, stirring continued for 30 min.

### 2.5. Droplet Size Measurements, Loading Capacity, UV-Visible Light Absorption Spectrum and Fluorescence Spectrum

The droplet size distributions of the ILQ-NEs were determined using Brookhaven 90 Plus PALS dynamic light scattering (DLS) equipment (Brookhaven Instruments Corporation, Holtsville, NY, USA) at 25 °C. The refractive index was set at 1.33. The mean droplet diameter and polydispersity index (PDI) were calculated from the droplet diameter distribution. All of the samples were diluted at a ratio of 1:50 with PBS (pH 7.4). The droplet size and distributions were measured immediately after the ILQ-NEs were prepared. The PDI values represent a method for describing the narrowness of the droplet diameter distribution, with small values representing a narrow distribution. The loading capacity was calculated as the percentage of the ILQ weight divided by the sum weight of the Cremophor^®^ EL and Labrafil^®^ M 1944 CS. The blank nanoemulsions used in the UV-visible light absorption spectra and fluorescence emission spectra measurements were prepared in the same way as the ILQ-NEs, but no ILQ was used. Moreover, SF-ILQ-Nes (sonication-free isoliquiritigenin nanoemulsions) were prepared in the same way as the ILQ-NEs, but no sonication was carried out. The absorption spectra were obtained on a UV-visible spectrophotometer (A360, Aoyi Instruments Co., LTD, Shanghai, China) at 25 °C. Fluorescence spectra were obtained using cuvettes with a 1 cm path length and a slit width of 10 nm at 298 K on a Fluorescence-4600 spectrometer (Hitachi High-technologies Corporation, Tokyo, Japan).

### 2.6. Transmission Electron Microscopy (TEM)

The morphology of the ILQ-NE droplets was observed via TEM. To achieve this, 10 μL samples of ILQ-NE were dropped onto a carbon-coated copper grid, and photographed by TEM (JEOL-2100F, JEOL Ltd., Tokyo, Japan) after the sample had dried naturally.

### 2.7. Physical Stability of ILQ-NE

The physical stability of ILQ-NE after long-term storage at 4 °C and in the physiological environment was investigated by mean droplet diameter determinations and visual appearance observations, as well as changes in the UV-visible light absorption spectrum. For the storage stability of ILQ-NE at 4 °C, droplet diameter measurements were performed every seven days during the 56-day storage period. All of the samples were diluted 50 times with PBS (pH 7.4) and then measured. To investigate their stability in the physiological environment, the ILQ-NEs were diluted at a ratio of 1:50 in PBS (pH 7.4), in physiological saline or in RPMI 1640 medium (containing 10% FBS), and were stored at 37 °C in incubators (GNP-9080BS-III, Zhongke Meiling Cryogenic Technology Co., LTD, Shanghai, China). The mean droplet diameters and the absorbance variations were recorded after storage for 0, 2, 4, 8, 12, 24, or 48 h.

### 2.8. Chemical Stability of ILQ-NE

ILQ-NE and free ILQ (4 mg ILQ in 2 mL PBS containing 2% Tween 80) were diluted to the same concentration (0.01 mg mL^−1^) using PBS, and then 40 mL diluted samples were placed in fixed positions in ultra-clean tables (McB-1300v, Xinmiao Medical Instrument Manufacturing Co., LTD, Shanghai, China) and subjected to UV irradiation at the same time. The source, power, power density, and wavelength of the UV irradiation were PHILIPS TUV, 8 W, >400 W/m^2^ and 253.7 nm, respectively. After UV irradiation for 0, 20, 40, or 60 min, the samples were collected and the ILQ contents were evaluated using a UV-visible spectrophotometer (A360, Aoyi Instruments Co., LTD, Shanghai, China) at 377 nm. In addition, the UV-visible light absorption spectrum of ILQ-NE during long-term storage at 4 °C was also recorded.

### 2.9. In Vitro Release Study

The in vitro release of free ILQ or ILQ-NE was evaluated via the dialysis–diffusion method as previously reported with slight modification [3]. Free ILQ (4 mg ILQ in 2 mL PBS containing 2% Tween 80) or ILQ-NE (2 mL, containing an equivalent amount of ILQ) was added to a dialysis bag with regenerated cellulose membranes (Mw = 3500 Da). Then, the dialysis bag was put into 200 mL of release media (PBS, pH 7.4, containing 1% Tween 80) in temperature conditions of 37 ± 0.5 °C and at a stirring rate of 100 r min^−1^. After stirring for 0, 6, 12, 24, or 36 h, dialysate (2 mL each) was removed and immediately replaced with 2 mL of the fresh receiving medium. The ILQ content in the medium was determined using a UV-visible spectrophotometer (A360, Aoyi Instruments Co., LTD, Shanghai, China).

### 2.10. Cell Culture and Cytotoxicity Test

Cells (4T1 cells) were obtained from Cell Bank, Chinese Academy of Sciences (Shanghai, China). The cells were cultivated in RPMI 1640 medium containing 1% penicillin–streptomycin solution and 10% FBS at 37 °C in an incubator containing 5% CO_2_.

To compare the cancer cell-killing effects of the different ILQ formulations, the in vitro cytotoxicity of the ILQ-NE, ILQ suspension (4 mg ILQ dissolved in 2 mL PBS containing 2% Tween 80), and ILQ in DMSO (2 mg ILQ dissolved in 1 mL DMSO) was tested on 4T1 cells using the CCK-8 method. The concentration of the ILO-NE, the ILQ suspension, or the ILQ in DMSO was varied by dilution with RPMI 1640 medium. First, cells were seeded onto 96-well plates at a density of 1 × 10^4^ cells well^−1^ and were incubated in a 37 °C incubator containing 5% CO_2_. After 24 h, the cells were treated with RPMI 1640 medium or medium containing the ILQ-NE, ILQ suspension or ILQ in DMSO (5, 10, and 20 μg mL^−1^, diluted by 1640 medium), and incubated for 24 h. Subsequently, the medium was replaced with 100 μL of 1640 medium containing 10 μL CCK-8. After incubation at 37 °C for 2 h, the absorbance at 450 nm was recorded using a Microplate Reader (ELX800, Bio Tek instruments, Inc., Highland Park, IL, USA). The effect of the formulations on the cell morphology was investigated using an inverted light microscope (ICX41, Carl Zeiss Microscopy GmbH, Germany) after 24 h of incubation with the ILQ-NE, ILQ suspension or ILQ in DMSO (ILQ 20 μg mL^−1^).

To evaluate the influence of different ILQ vehicles on the cancer cell-killing effect, the cytotoxicity of DMSO (1 mL DMSO), the blank suspension (2 mL PBS containing 2% Tween 80), or blank NE (prepared in the same way as the ILQ-NEs but no ILQ was used) were tested. The blank suspension was prepared in the same way as the ILQ suspension but ILQ was not used. Initially, the 4T1 cells were planted into 96-well plates at a density of 1 × 10^4^ cells well^−1^ and incubated at 37 °C in 5% CO_2_ for 24 h. Afterwards, the cells were co-incubated with DMSO, blank suspension, or blank NE with the same excipient concentrations as the corresponding ILQ formulations diluted to ILQ concentration of 20 μg mL^−1^ for 24 h. Subsequently, the liquid was sucked out, and 100 μL of 1640 medium containing 10 μL of CCK-8 was added to each well. After incubation at 37 °C for 2 h, the absorbance at 450 nm was determined using a Microplate Reader (ELX800, Bio Tek instruments, Inc., Highland Park, IL, USA).

### 2.11. Cellular Uptake Test

To investigate the cellular uptake of different ILQ formulations, first, the 4T1 cells were seeded onto 96-well plates at a density of 1 × 10^4^ cells well^−1^ and incubated in an incubator containing 5% CO_2_ at 37 °C. After 24 h, the cells were incubated with RPMI 1640 medium or medium containing ILQ-NE, ILQ suspension or ILQ in DMSO (ILQ 20 µg mL^−1^) for 24 h. After that, the fluorescence images were obtained with an inverted fluorescence microscope (ICX41, Carl Zeiss Microscopy GmbH, Jena, Germany). The excitation wavelength and emission wavelength of the inverted fluorescence microscope were set as 385 nm and 450 nm, respectively.

### 2.12. Statistical Analysis

All of the results are expressed as the mean value ± standard deviation (SD). Data were analyzed with SPSS software (edition 16.0, SPSS, Inc., Chicago, IL, USA). The independent-samples *t*-test followed by Levene’s Test for equality of variances was used to compare two mean values. One-way analysis of variance (ANOVA) analysis followed by Levene’s Test for equality of variances as well as Bonferroni’s multiple comparison test was used to compare three or more mean values. The accepted level of significance was *p* < 0.05.

## 3. Results and Discussion

### 3.1. Solubility Studies

**Figure 2:**
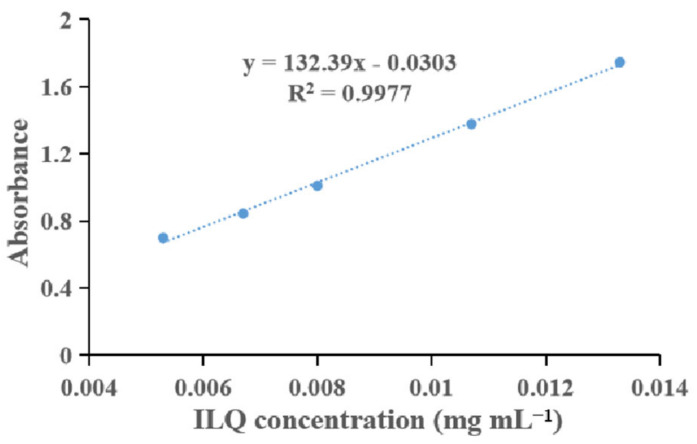
Standard curve for the relationship between light absorbance and ILQ concentration. ILQ, isoliquiritigenin.

First, the surfactant and oil types for the ILQ nanoemulsions were determined by screening high ILQ solubility surfactants and oil phase excipients. As shown in Figure 2, there was a satisfactory linear relationship (R^2^ = 0.9977) between the light absorbance and ILQ concentration, which indicated the suitability of the absorbance for evaluating the ILQ content. The solubility of ILQ at 25 °C in various excipients is shown in Table 1. Among the oils screened, Labrafil^®^ M 1944 CS showed the highest solubility (703.32 ± 0.0164 mg g^−1^). Meanwhile, ILQ demonstrated its highest solubility in Cremophor^®^ EL (664.33 ± 0.0065 mg g^−1^) among the tested surfactants. Cremophor^®^ EL creates a strong spatial repulsive force that prevents droplet aggregation and has low toxicity [23]. In this context, Labrafil^®^ M 1944 CS was chosen as the oil phase, and Cremophor^®^ EL was selected as the surfactant.

### 3.2. Optimization of ILQ-NE Preparation

#### 3.2.1. Effect of Stirring Speed on the Appearance, Droplet Size and Storage Stability

**Figure 3:**
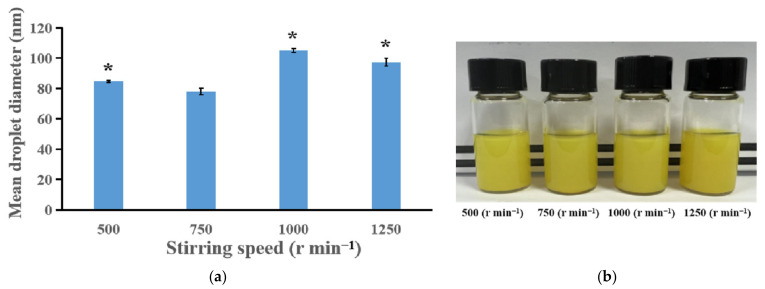
(**a**) The effects of stirring speed on the droplet size of ILQ nanoemulsions prepared by the SPIC method. (**b**) Appearance of ILQ nanoemulsions prepared at different stirring speeds. Values are expressed as mean ± SD (*n* = 3). * *p* < 0.05 in comparison with the 750 r min^−1^ group.

In fixed conditions such as the oil phase (Labrafil^®^ M 1944 CS, 0.18 g), surfactant (Cremophor^®^ EL, 0.12 g) and ILQ (24 mg), the effect of stirring speed on the droplet diameters of the nanoemulsions prepared via the SPIC method was investigated. Generally, the droplet diameter first decreased and then increased as the stirring speed increased within the range of 500–1250 r min^−1^ (Figure 3a). A similar trend was observed in a study focusing on the preparation of nanoemulsions using a spontaneous emulsification method, a low-energy method whose mechanism is similar to that of the PIC method [24]. These results suggest that mild mixing of the organic and aqueous phases could promote the formation of small droplets through low-energy methods. The smallest droplets were created at 750 r min^−1^. As seen from Figure 3b, the emulsions prepared at various stirring speeds showed a turbid appearance. Moreover, after storage for one month, precipitation was observed in all of the emulsion samples. These indicated that further optimization concerning other preparation parameters was needed for production of nanoemulsions with tiny droplets and high stability.

#### 3.2.2. Effect of Ultrasonic Depth on the Droplet Size, Appearance and Storage Stability

**Figure 4:**
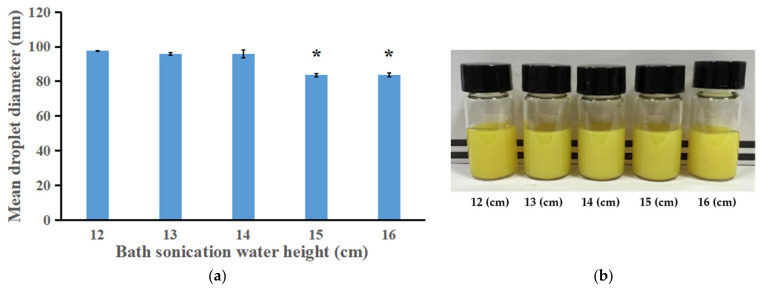
(**a**) The effect of bath sonication water height on the droplet size of ILQ nanoemulsions prepared by the SPIC method. (**b**) Appearance of ILQ nanoemulsions prepared at different bath sonication water heights. Values are expressed as mean ± SD (*n* = 3). * *p* < 0.05 in comparison with the 12 cm group.

To investigate the effect of ultrasonic depth, i.e., the height of the water in the ultrasonic cleaning machine, on the formation of small droplets using the SPIC method, small vials containing organic phases were partially (1.5 cm was under water) placed into the water, and the height of the water in the ultrasonic cleaning machine was varied. As shown in Figure 4a, significantly smaller mean droplet diameters were obtained when the bath sonication water heights were set at 15 cm or 16 cm, in comparison with other water heights. Emulsions that were prepared at different heights showed a turbid appearance (Figure 4b). After storage for one month, the nanoemulsions prepared at the water height of 15 cm exhibited less sediment than that produced at the 16 cm height. Therefore, 15 cm was selected as the optimal ultrasonic water height and was used in the subsequent experiments. The effects of ultrasonic depth on the droplet size and storage stability might be attributed to the difference in ultrasonic power created by variations in water height.

#### 3.2.3. Effect of Sonication Time on the Droplet Size, Appearance and Storage Stability

**Figure 5:**
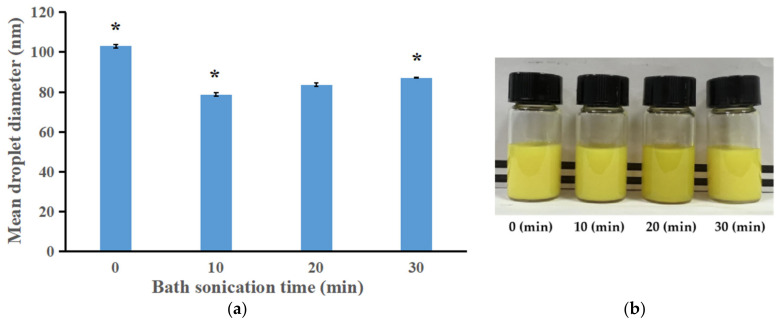
(**a**) The effects of bath sonication time on the droplet size of ILQ nanoemulsions prepared by the SPIC method. (**b**) Appearance of freshly-prepared ILQ nanoemulsions produced at different bath sonication durations (upper panel) and the appearance of ILQ nanoemulsions at 10 min and 20 min after storage for 30 days. Values are expressed as mean ± SD (*n* = 3). * *p* < 0.05 in comparison with the 20 min group.

As shown in Figure 5a, as the duration of the bath sonication treatment increased from 0 to 10 min, the droplet size decreased from 103.03 to 78.76 nm. However, when the bath sonication time increased to 20 and 30 min, the droplet size increased to 83.61 nm and 87.20 nm, respectively. The same trend was observed in Shabnam Asadinezhad et al.’s research on the influence of the bath sonication time on the droplet size in nanoemulsion [20]. Another study on sacha inchi oil nanoemulsion preparation also showed that under specific ultrasonic amplitude, the droplet size first decreased and then increased as the sonication time increased [25]. The 10 min and 20 min groups presented smaller droplet diameters in comparison with other groups. Figure 5b (upper panel) presents the appearance of freshly prepared nanoemulsions. A turbid appearance was observed regardless of the sonication time. However, after storage for one month (Figure 5b lower panel), an obviously higher quantity of precipitate was observed in the nanoemulsion from the 10 min group than in the nanoemulsion from the 20 min group, indicating that the ILQ nanoemulsions produced with 20 min sonication had higher stability. Therefore, 20 min was selected as the optimal sonication time and was used in the subsequent experiments.

#### 3.2.4. Effect of ILQ Concentration on the Droplet Size, Appearance and Storage Stability

**Figure 6:**
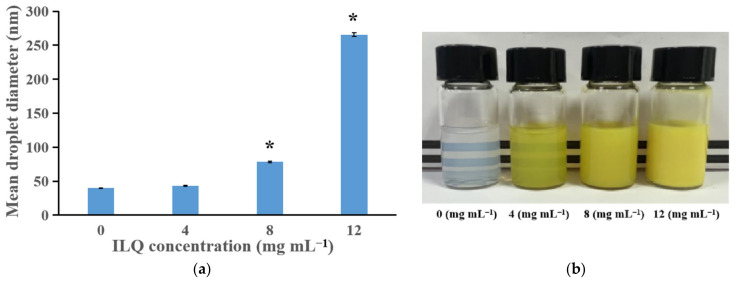
(**a**) The effect of ILQ concentration on the droplet size of ILQ nanoemulsions prepared by the SPIC method. (**b**) Appearance of ILQ nanoemulsions with different ILQ contents. Values are expressed as mean ± SD (*n* = 3). * *p* < 0.05 in comparison with the 0 mg mL^−1^ group.

As shown in Figure 6a, the droplet diameter of ILQ nanoemulsions was strongly dependent on the ILQ content. Minor droplets (d < 50 nm) were obtained in the nanoemulsions with ILQ contents equal to 4 mg mL^−1^ (i.e., 12 mg ILQ in 3 mL system), while larger droplets were obtained at higher ILQ contents. As the ILQ concentration increased, the droplet size increased. Consistent with this research, it was previously reported that increasing the concentration of the cargo substance astaxanthin elicited droplet diameter growth [26]. In this research, the 4 mg mL^−1^ concentration of ILQ did not cause obvious increases in the droplet diameter (*p* > 0.05), and rendered a homogeneous and transparent appearance (Figure 6b). In addition, after one month of storage, the nanoemulsions with ILQ concentrations of 0 or 4 mg mL^−1^ showed no precipitation, while the ones with ILQ concentrations of 8 or 12 mg mL^−1^ exhibited sedimentation, with the 12 mg mL^−1^ ILQ group bearing larger precipitate quantities than the 8 mg mL^−1^ ILQ group.

### 3.3. Physicochemical Properties of ILQ-NE

**Figure 7:**
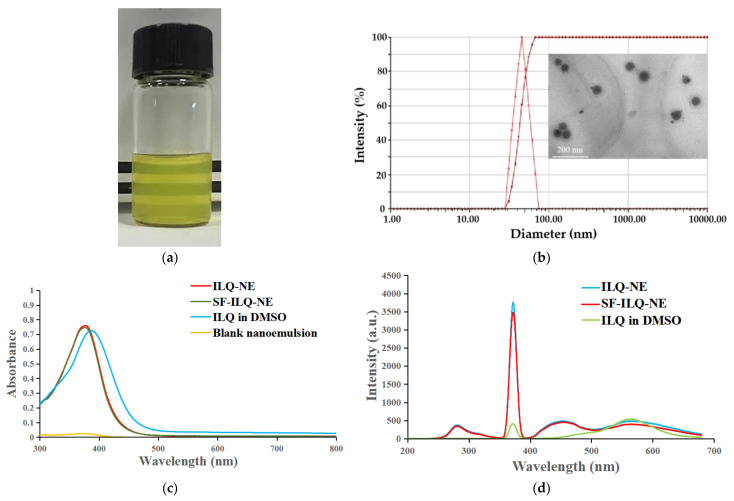
Physicochemical properties of ILQ-NE. (**a**) The appearance of ILQ-NE (ILQ concentration 4 mg mL^−1^). (**b**) The droplet size distribution of ILQ-NE (ILQ concentration 4 mg mL^−1^). Inset: transmission electron microscopic image of ILQ-NE. Water was used as the aqueous phase to avoid the influence of PBS on the photograph. (**c**) Absorption spectra of ILQ-NE, SF-ILQ-NE, and ILQ in DMSO, and blank nanoemulsion. (**d**) Fluorescence spectra of ILQ-NE, SF-ILQ-NE, and ILQ in DMSO. ILQ-NE, isoliquiritigenin nanoemulsion. SF-ILQ-NE, sonication-free isoliquiritigenin nanoemulsion. DMSO, dimethyl sulfoxide.

Ordinary microscale emulsions usually appear as opaque or turbid because of strong multiple light scattering. However, nanoemulsions with droplet sizes well below 100 nm typically exhibit a transparent appearance, because the sizes of the droplets are much smaller than the wavelengths of visible light (400–800 nm), leading to relatively weak light scattering [27]. ILQ-NE (4 mg mL^−1^) presented a transparent, homogeneous, and yellow appearance (Figure 7a), suggesting that the droplet size was below 100 nm. Because of its chalcone-type structure, the aqueous solubility of ILQ is very low. It has been reported that the solubility of ILQ in water is merely 3.74 μg mL^−1^ [6]. In contrast, our nanoemulsion system increased the solubility of ILQ to 4 mg mL^−1^, which was more than 1000 times that of the intrinsic solubility of ILQ. The loading capacity was 4%. Compared with the ILQ concentration of previously reported polymeric micelles (0.874 mg mL^−1^), the ILQ concentration of ILQ-NE (4 mg mL^−1^) is higher [6]. The DLS measurement results demonstrated that the mean droplet diameter of ILQ-NE was 44.10 ± 0.28 nm (Figure 7b), which was consistent with the visual appearance. This is significantly smaller than the droplet sizes of recently reported ILQ nanosuspensions (238.1 ± 4.9 nm and 354.1 ± 9.1 nm) and lipid-polymer hybrid nanoparticles (137.2 ± 2.6 nm) [5,9]. The small droplet size could facilitate the biomedical application of ILQ via mechanisms such as promoting its absorption to and permeation through the intestinal membrane [28]. The PDI was 0.098 ± 0.016 (Figure 7b), indicating a narrow distribution which was important for preventing droplet growth caused by Ostwald ripening [29]. The TEM image revealed the spherical morphology of ILQ-NE droplets (Figure 7b inset). The droplet size obtained by the TEM photograph was consistent with the DLS measurement results.

The UV-visible light absorption spectrum and fluorescence emission spectrum for ILQ-NE are shown in Figure 7c,d, respectively. As shown in Figure 7c, the blank nanoemulsion (containing no ILQ) presented hardly any absorption throughout the 300–800 nm range, while ILQ-NE showed a strong absorption peak that was similar to the peak of ILQ in the DMSO sample. This indicated that ILQ was successfully encapsulated into our nanoemulsion system. More importantly, we compared the UV-visible light absorption spectra of ILQ-NE and SF-ILQ-NE, the nanoemulsion that was prepared following the same methods as ILQ-NE but without sonication. These two types of nanoemulsions exhibited identical absorption curves, demonstrating that the sonication used in our SPIC method did not cause damage to environment-sensitive ILQ when it effectively decreased the droplet size. The fluorescence emission spectra presented in Figure 7d verifies the conclusions obtained from the UV-visible light absorption spectra results. ILQ-NE exhibited the same characteristic peak of ILQ as DMSO, indicating successful ILQ encapsulation. Meanwhile, the emission spectra of ILQ-NE and SF-ILQ-NE were almost the same, which indicated that the sonication in the SPIC method did not damage ILQ. In addition, the fluorescence emission intensity of ILQ-NE was 9.38 times of ILQ in DMSO at 372 nm, demonstrating that nanoemulsion incorporation greatly enhanced the fluorescence emission. This suggests the potential of our nanoemulsion system to boost the fluorescence emissions of the encapsulated dye and to benefit biomedical fluorescence imaging [30].

### 3.4. Physical Stability of ILQ-NE

**Figure 8:**
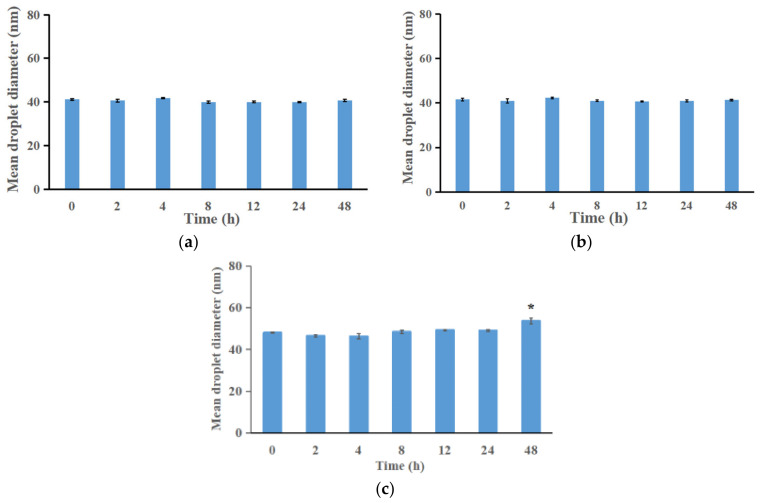
(**a**) Changes in ILQ-NE droplet size in PBS during storage for 48 h at 37 ± 0.5 °C. (**b**) Change in ILQ-NE droplet size in physiological saline during storage for 48 h at 37 ± 0.5 °C. (**c**) Change in ILQ-NE droplet size in RPMI 1640 medium (containing 10% FBS) during storage for 48 h at 37 ± 0.5 °C. Values are expressed as mean ± SD (*n* = 3). * *p* < 0.05. ILQ-NE, the optimized ILQ nanoemulsion.

**Figure 9:**
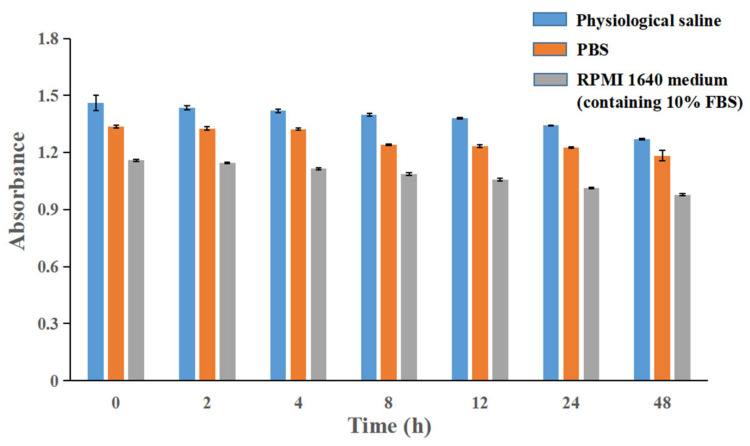
Change in ILQ-NE absorbance in PBS, physiological saline and RPMI 1640 medium (containing 10% FBS) during storage for 48 h at 37 ± 0.5 °C. Values are expressed as mean ± SD (*n* = 3).

**Figure 10:**
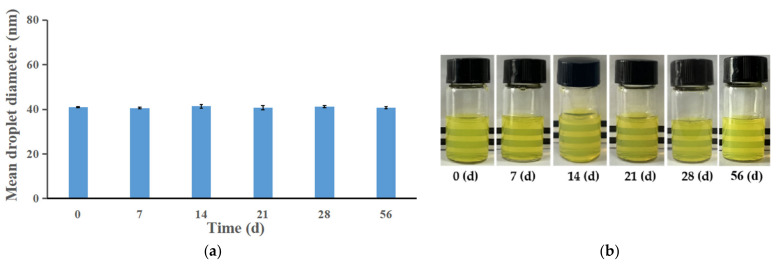
(**a**) The change in droplet size of ILQ-NEs during storage at 4 °C for 56 days. (**b**) The appearance of ILQ-NEs preserved at 4 °C and stored for 0, 7, 14, 21, 28, or 56 days. Values are expressed as mean ± SD (*n* = 3). ILQ-NE, the optimized ILQ nanoemulsion.

The physical stability of ILQ-NE was investigated both under the physiological environment (Figure 8 and Figure 9) and during long-term storage at 4 °C (Figure 10).

#### 3.4.1. Physical Stability of ILQ-NE in Physiological Environment

To evaluate the stability of ILQ-NE in the physiological environment, ILQ-NE in pH 7.4 PBS, physiological saline or RPMI 1640 medium containing 10% FBS was stored at 37 °C for 48 h. During storage, the appearance of the system was checked, the droplet size was determined by DLS measurements, and the absorbance was measured using a UV-visible spectrophotometer. The absorbance was investigated in order to monitor possible precipitation which could not be detected by appearance check or DLS measurements. During storage at 37 °C for 48 h, neither sedimentation nor variation in the droplet diameter were detected (*p* > 0.05) in pH 7.4 PBS (Figure 8a) or in physiological saline (Figure 8b), which indicated the satisfactory stability of ILQ-NE in pH 7.4 PBS and in physiological saline. Although the droplet size slightly increased (*p* < 0.05) in the RPMI 1640 medium (containing 10% FBS), the diameter was still well below 100 nm (Figure 8c), which might keep the advantage originated from nano size of ILQ-NE in biomedical applications. In addition, the absorbance results (Figure 9) showed that the ILQ remained up to 88.6%, 87.0%, and 84.3% in PBS, physiological saline, and RPMI 1640 medium containing 10% FBS, respectively. The small reduction in ILQ content should originate from the ILQ degradation rather than precipitation after storage at 37 °C for 48 h, since ILQ is easily degraded when the temperature is elevated. Generally speaking, the absorbance results indicated that the position of ILQ was within the dispersion rather than within precipitation, which implied the reliability of the DLS results. In a word, the results above suggest that the nanoemulsion incorporation might facilitate the biomedical application of ILQ via elongating the circulation time of ILQ.

#### 3.4.2. Physical Stability of ILQ-NE during Long-Term Storage at 4 °C

The physical stability of the ILQ-NE during storage at 4 °C was evaluated by droplet diameter measurements and visual appearance. The mean droplet diameter of ILQ-NE remained almost the same throughout the 56-day storage period at 4 °C (*p* > 0.05, Figure 10a). Meanwhile, no creaming or aggregation was observed, and the ILQ-NE remained clear and transparent during the 56-day storage period (Figure 10b). The electrostatic repulsion and steric repulsion among the oil droplets covered by surfactants are two important mechanisms underlying the storage stability of nanoemulsion systems [31]. On the one hand, the surfactant in ILQ-NE, Cremophor^®^ EL, is a non-ionic surfactant, and it consequently created a very faint electrostatic repulsive interaction [26]. On the other hand, the large hydrophilic head groups (polyoxyethylene chains) of Cremophor^®^ EL can form relatively thick interfaces that create intense and long range steric repulsion to avoid the droplet aggregation being observed [32]. In summary, steric repulsion may be the primary stabilizing mechanism for ILQ-NE. Because of the strong steric repulsion, ILQ-NE remained clear (Figure 10b) with no instability phenomena such as creaming and aggregation after storage at 4 °C for 56 days. This is of great significance for the transportation and application of ILQ-NE.

### 3.5. Chemical Stability of ILQ-NE

#### 3.5.1. Chemical Stability of ILQ-NE in Comparison with Free ILQ

**Figure 11:**
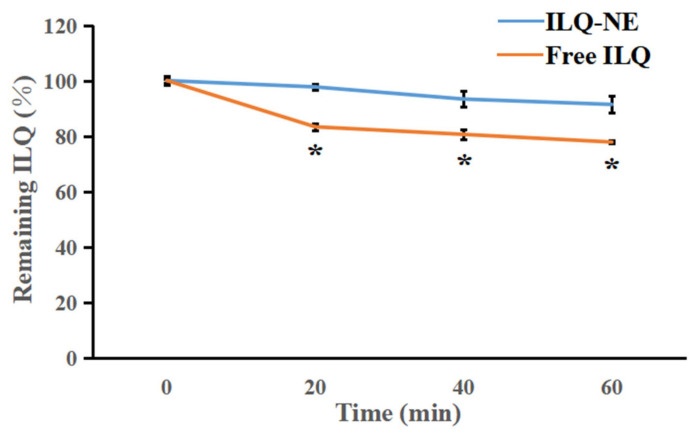
The change in ILQ content for ILQ-NE or free ILQ after ultraviolet irradiation for 0, 20, 40, or 60 min. Values are expressed as mean ± SD (*n* = 3). * *p* < 0.05, in comparison with corresponding free ILQ group. ILQ-NE, the optimized ILQ nanoemulsion. ILQ, isoliquiritigenin.

The structure of ILQ falls into the same category of flavonoids, which usually have poor chemical stability [33]. To investigate the chemical stability of ILQ-NE in comparison with free ILQ (4 mg ILQ in 2 mL PBS containing 2% Tween 80), the change in ILQ content in ILQ-NE or free ILQ during UV irradiation at 25 °C was recorded for up to 60 min (Figure 11). UV irradiation usually exacerbates the oxidation and degradation of bioactive ingredients. In this research, the concentration of ILQ in the ILQ-NE decreased more slowly than that in free ILQ (*p* < 0.05), indicating that nanoemulsion formulations had a superior protective effect compared with aqueous dispersions. The improved chemical stability may be ascribed to the protective layer formed by the oil and surfactant molecules, and might promote the biomedical application of ILQ.

#### 3.5.2. Chemical Stability of ILQ-NE during Long-Term Storage at 4 °C

**Figure 12:**
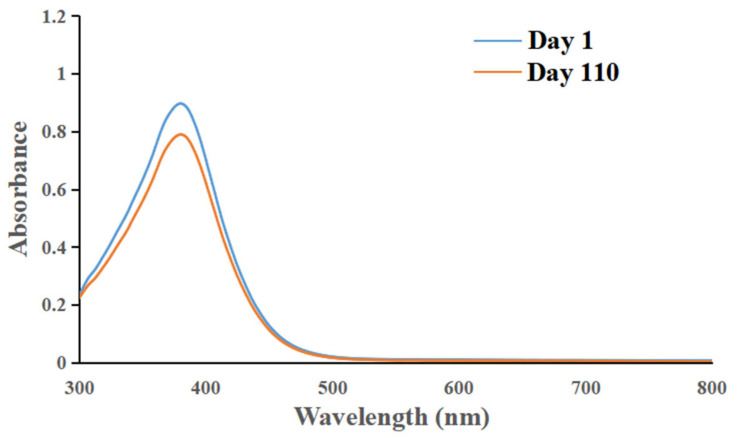
The change in ultraviolet-visible light absorption spectrum of ILQ-NEs during storage at 4 °C for 110 days. ILQ-NE, the optimized ILQ nanoemulsion.

The chemical stability of ILQ-NE during storage at 4 °C was evaluated by the variation in the UV-visible light absorption spectrum. The UV-visible light absorption spectrum of ILQ-NE remained almost the same throughout the 110-day storage period at 4 °C. As is shown in Figure 12, more than 88% of the ILQ was remained after 110 days of storage at 4 °C.

### 3.6. Drug Release Profile In Vitro

**Figure 13:**
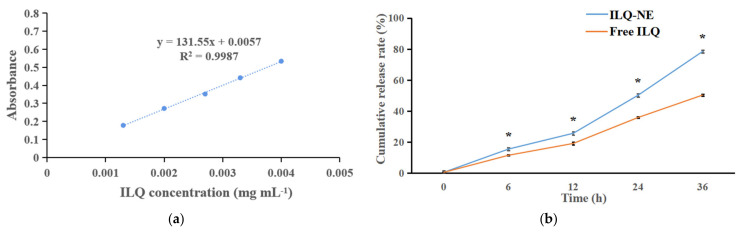
(**a**) Standard curve for the relationship between absorbance and ILQ concentration. (**b**) Cumulative release rate for ILQ-NE or free ILQ in PBS (pH 7.4). Values are expressed as mean ± SD (*n* = 3). * *p* < 0.05, in comparison with corresponding free ILQ group. ILQ-NE, the optimized ILQ nanoemulsion. ILQ, isoliquiritigenin.

Because of its poor aqueous solubility, ILQ has a low dissolution rate through the intestinal membrane and low bioavailability, which impedes its further in vivo application. Therefore, in this research, the release profiles of ILQ-NE in PBS (pH 7.4, containing 1% Tween80) in comparison with free ILQ, were investigated using the dialysis–diffusion method. As shown in Figure 13a, there was a good linear relationship (R^2^ = 0.9987) between the absorbance and ILQ concentration, indicating the suitability of absorbance for evaluating the ILQ content. As presented in Figure 13b, free ILQ exhibited a poor cumulative release in pH 7.4 PBS at the end of 36 h (50.30 ± 0.67%). In comparison, the cumulative release of ILQ-NE in PBS (pH 7.4) at 36 h reached 78.43 ± 0.71%, demonstrating that only the ILQ packaged in nanoemulsion formulations could acquire a high dissolution rate. Under neutral (pH = 7.4) condition, the cumulative release of ILQ-NE is significantly higher than the recently reported mesoporous silica nanoparticles (23.97 ± 1.35%) [10]. This enhanced ILQ release might be attributed to the small size of the ILQ-NE droplets, which facilitated the permeation process through the dialysis bag. The results are similar to those reported in earlier studies [3]. The improved ILQ solubility, and consequently enhanced release, might promote the permeation of ILQ through the intestinal membrane, and increase the bioavailability of ILQ.

### 3.7. Assessment of Cytotoxicity

**Figure 14:**
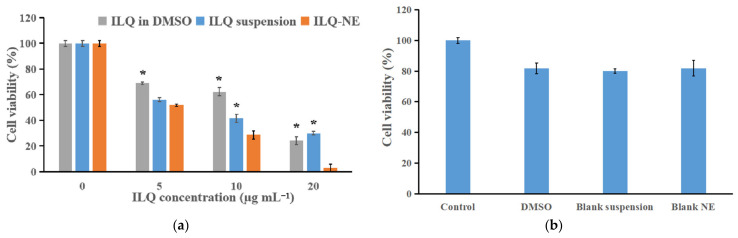
Cytotoxicity investigation of ILQ-NE. (**a**) Viability of 4T1 cells treated with ILQ-NE, ILQ suspension, or ILQ in DMSO at 0, 5, 10 and 20 μg mL^−1^. (**b**) The influence of blank NE, DMSO, and blank suspension on 4T1 cells at 20 μg mL^−1^. Values are expressed as mean ± SD (*n* = 3). * *p* < 0.05, in comparison with corresponding ILQ-NE group. ILQ-NE, the optimized ILQ nanoemulsion. ILQ, isoliquiritigenin.

**Figure 15:**
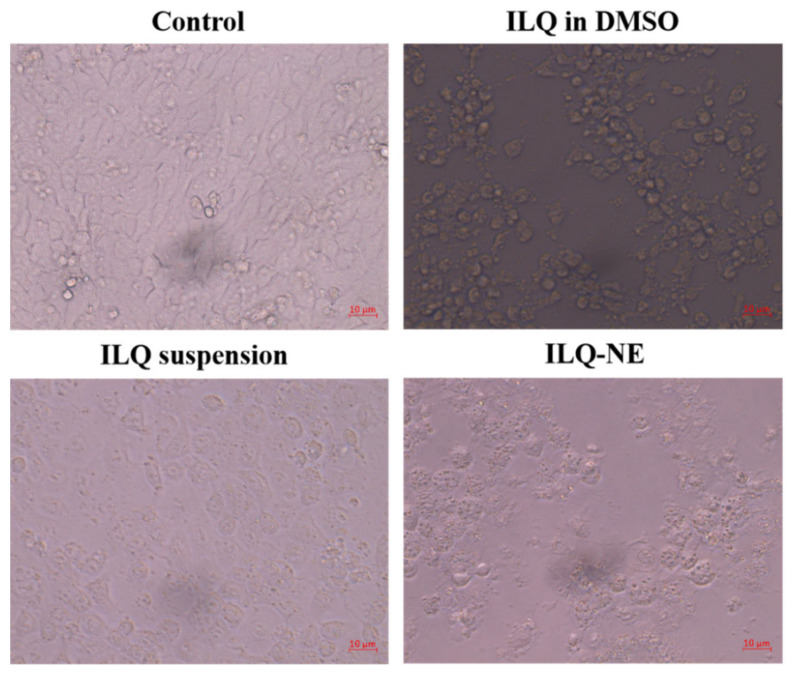
The 4T1 cell morphologies under different treatment conditions. ILQ-NE, the optimized isoliquiritigenin nanoemulsion. DMSO, dimethyl sulfoxide.

First, in order to compare the cytotoxicity of ILQ-NE and that of free ILQ (including ILQ suspension and ILQ in DMSO), the CCK-8 assay was used to test the cytotoxicity of ILQ-NE, ILQ suspension, and ILQ in DMSO against 4T1 cancer cells. The 4T1 cells were selected for the in vitro studies because the characteristics of these cells well represented breast cancer, and ILQ could inhibit breast cancer via mechanisms such as vascular endothelial growth factor-vascular endothelial growth factor receptor-2 (VEGF-VEGFR2) pathway and NF-κB pathway [1,9]. The 4T1 cells were treated with culture medium or a series of concentrations of ILQ in DMSO, ILQ suspension, or ILQ-NE (at ILQ concentration 5, 10, or 20 μg mL^−1^) for 24 h to calculate the percentage cell viability. Figure 14a indicated that the percentage cell viabilities of pure ILQ (either ILQ in DMSO or ILQ suspension) and ILQ-NE were dose-dependent. Compared with the ILQ suspension and ILQ in DMSO, the inhibitory effect of ILQ-NE was stronger when the ILQ concentration was at 10 or 20 μg mL^−1^. Moreover, the morphologies of 4T1 cells after incubation for 24 h with ILQ-NE, ILQ suspension or ILQ in DMSO at ILQ concentration 20 μg mL^−1^ were clearly observed by an inverted microscope. As shown in Figure 15, the control group (4T1 cells treated with merely 1640 culture medium) presented elongated shape, indicating normal cell morphology. Free ILQ formulations (ILQ in DMSO or ILQ suspension) group exhibited mild cell morphology change from elongated to spherical shape. In contrast, cells treated with ILQ-NE exhibited magnificent morphology alteration, as most of the cells were spherical and appeared to contain small vesicles or particles, which implied the fate of cell breaking down. The morphological observations were consistent with the cell viability assay results, and indicated the superior toxicity of ILQ-NE to cancer cells in comparison with free ILQ formulations.

Furthermore, to demonstrate that the superior cytotoxicity of ILQ-NE originated from the ILQ nanoencapsulation rather than from the excipients (DMSO, blank suspension components, and blank NE components) themselves, the cytotoxicity of blank NE, DMSO, and the blank suspension (with the same excipient concentrations as the corresponding ILQ formulations diluted to ILQ concentration of 20 μg mL^−1^) to the 4T1 cells was investigated. Figure 14b showed that all of them had merely weak cytotoxicity to 4T1 cells, and that there was no significant difference in cell viability.

In summary, these cytotoxicity test results indicate that the nanoencapsulation of ILQ with our nanoemulsion system greatly improved the cancer cell-killing ability of ILQ. Consistently, recent research showed that other drug carriers such as composite hydrogels may contribute to anticancer activities [34,35].

### 3.8. Cellular Uptake Test

**Figure 16:**
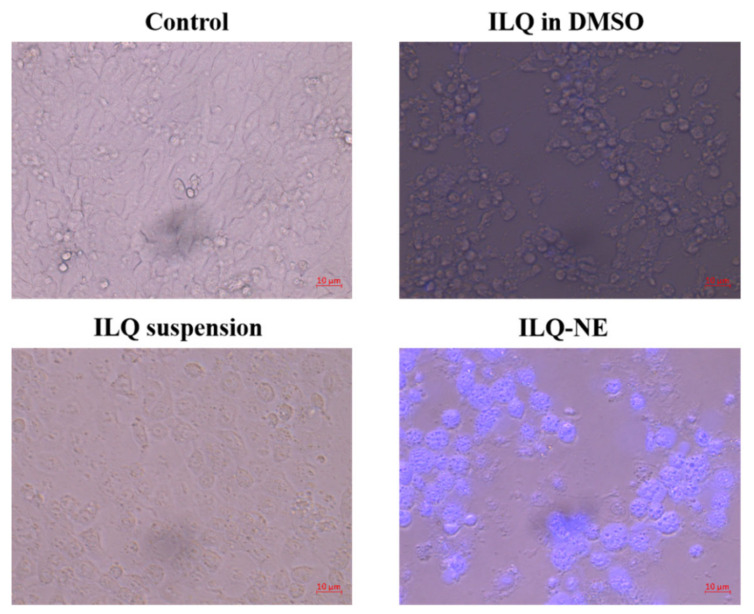
The 4T1 cellular uptake under different treatment conditions. ILQ-NE, the optimized isoliquiritigenin nanoemulsion. DMSO, dimethyl sulfoxide.

To further investigate the mechanism underlying the superior cytotoxicity of ILQ-NE in comparison with free ILQ formulations, the ILQ uptake by 4T1 cells was evaluated by the fluorescence generated from ILQ using an inverted fluorescence microscope (Figure 16). The imaging of different treatment groups was performed in the same experimental conditions such as emissive light power and power density. The cells treated with culture medium (i.e., control group) showed no fluorescence at all. ILQ in the DMSO group and ILQ suspension group exhibited little fluorescence, perhaps because their large droplet sizes impeded the cellular internalization. In sharp contrast, strong blue fluorescence was observed in the ILQ-NE group, which demonstrated a large quantity of ILQ entered the cancer cells. This can be ascribed to the small droplet size of ILQ-NE which facilitated the permeability of ILQ through cancer cell membranes. These findings indicate that ILQ-NE may improve toxicity to cancer cells via facilitating the ILQ entrance into cells. The superior cytostatic effect of ILQ-NE compared with two free ILQ formulations may be attributed to stronger internalization of ILQ-NE into cells by phagocytosis, owing to its small droplet size [36].

## 4. Conclusions

In the present study, through stirring speed, bath sonication water height, bath sonication time, and ILQ concentration optimization ILQ-NE was successfully developed by applying the SPIC method. The ILQ-NE consisted of Labrafil^®^ M 1944 CS (oil), Cremophor^®^ EL (surfactant), ILQ, and PBS (pH = 7.4). The solubility of the ILQ-NE was improved ~1000 times more than the intrinsic solubility of ILQ. The ILQ-NE contained spherical droplets with a mean diameter of ~44 nm and a narrow size distribution. It enhanced the chemical stability and in vitro release rate of ILQ, and demonstrated satisfactory physical stability. Additionally, in comparison with free ILQ formulations, ILQ-NE more strongly enhanced the ability of ILQ to kill cancer cells, probably via stronger cellular uptake elicited by ILQ nanoencapsulation. In conclusion, ILQ-NE provides a new promising pharmaceutical candidate for cancer treatment and could facilitate the biomedical application of ILQ. In addition, the SPIC method might expand surfactant and oil choices and lower the surfactant concentration for the PIC method, while maintaining its merit of relatively low cost.

## Figures and Tables

**Table 1:** Solubility of isoliquiritigenin (ILQ) in different excipients. Values are expressed as mean ± SD (*n* = 3).

Excipients	Solubility (mg g^−1^)	Excipients	Solubility (mg g^−1^)
Peanut oil Canola oil	103.89 ± 0.0032 113.55 ± 0.0044	Tween 85 Tween 80	445.33 ± 0.0068 447.72 ± 0.0019
Corn oil Soybean oil	113.98 ± 0.0031 147.12 ± 0.0044	Tween 40 Tween 60	568.85 ± 0.0086 573.49 ± 0.0054
Labrafil^®^ M 1944 CS	703.32 ± 0.0164	Cremophor^®^ EL	664.33 ± 0.0065

## Data Availability

Not applicable.
